# Development of an autophagy-related gene expression signature for prognosis prediction in prostate cancer patients

**DOI:** 10.1186/s12967-020-02323-x

**Published:** 2020-04-07

**Authors:** Daixing Hu, Li Jiang, Shengjun Luo, Xin Zhao, Hao Hu, Guozhi Zhao, Wei Tang

**Affiliations:** 1 Department of Urology, The First Affiliated Hospital of Chongqing Medical University, No.1 Youyi Road, Yuan Jiagang, Yuzhong District, Chongqing, 400010 People’s Republic of China; 2 Department of Urology, The People’s Hospital of Nan Chuan, Chongqing, 408400 People’s Republic of China

**Keywords:** TCGA, GEO, Prostate cancer, Survival, Autophagy

## Abstract

**Background:**

Prostate cancer (PCa) is one of the most prevalent cancers that occur in men worldwide. Autophagy-related genes (ARGs) may play an essential role in multiple biological processes of prostate cancer. However, ARGs expression signature has rarely been used to investigate the association between autophagy and prognosis in PCa. This study aimed to identify and assess prognostic ARGs signature to predict overall survival (OS) and disease-free survival (DFS) in PCa patients.

**Methods:**

First, a total of 234 autophagy-related genes were obtained from The Human Autophagy Database. Then, differentially expressed ARGs were identified in prostate cancer patients based on The Cancer Genome Atlas (TCGA) database. The univariate and multivariate Cox regression analysis was performed to screen hub prognostic ARGs for overall survival and disease-free survival, and the prognostic model was constructed. Finally, the correlation between the prognostic model and clinicopathological parameters was further analyzed, including age, T status, N status, and Gleason score.

**Results:**

The OS-related prognostic model was constructed based on the five ARGs (FAM215A, FDD, MYC, RHEB, and ATG16L1) and significantly stratified prostate cancer patients into high- and low-risk groups in terms of OS (HR = 6.391, 95% CI = 1.581– 25.840, P < 0.001). The area under the receiver operating characteristic curve (AUC) of the prediction model was 0.84. The OS-related prediction model values were higher in T3-4 than in T1-2 (P = 0.008), and higher in Gleason score  > 7 than  ≤ 7 (P = 0.015). In addition, the DFS-related prognostic model was constructed based on the 22 ARGs (ULK2, NLRC4, MAPK1, ATG4D, MAPK3, ATG2A, ATG9B, FOXO1, PTEN, HDAC6, PRKN, HSPB8, P4HB, MAP2K7, MTOR, RHEB, TSC1, BIRC5, RGS19, RAB24, PTK6, and NRG2), with AUC of 0.85 (HR = 7.407, 95% CI = 4.850–11.320, P < 0.001), which were firmly related to T status (P < 0.001), N status (P = 0.001), and Gleason score (P < 0.001).

**Conclusions:**

Our ARGs based prediction models are a reliable prognostic and predictive tool for overall survival and disease-free survival in prostate cancer patients.

## Background

Autophagy is a process that maintains cellular homeostasis, which conducts damaged or defective intracellular components, also known as type II programmed cell death [1]. Abnormal autophagy function is closely associated with multiple diseases, such as immune disorders, neurodegenerative diseases, and cancers [2]. Several studies reported that autophagy could play a role in tumor progression or tumor suppression in different stages of cancers [3, 4]. However, the role of autophagy in tumorigenesis is still rudimentary.

Prostate cancer (PCa) is a common malignancy of the urinary system and the second cause of cancer-related death of males in western developed countries [5]. In China, the annual incidence of PCa was more than 60,000 cases and 26,600 patients who succumbed to PCa in 2015 [6]. The majority of early-stage PCa patients have an excellent prognosis with a low mortality rate [7]. However, there are still a large number of PCa patients who develop the resistance to androgen deprivation therapy (ADT) and become castration-resistant PCa (CRPC), which results in a short survival time [8].

The relationship between autophagy and multiple biological processes of prostate cancer has been previously reported [9]. For instance, in the early stage of PCa, autophagy may increase tumor cell death. However, elevated autophagy promotes prostate cancer invasion and progression and reduces the damage of chemotherapy drugs in the late stage. Cao et al. showed that the induction of autophagy might increase susceptibility to radiation in prostate cancer cell lines [10, 11]. However, large-scale gene expression signature has rarely been used to investigate the association between autophagy and prognosis in prostate cancer. To better understand the impact of tumor genetic composition on clinical outcomes, the Cancer Genome Atlas (TCGA) database has been established for discovering gene signatures.

There are many research and prognostic models based on gene expression profiles in prostate cancer, such as lncRNAs and miRNAs [12]. Nevertheless, prognostic models for autophagy-related genes have not been reported. In this study, we used gene expression microarray data obtained from TCGA to develop autophagy-related gene expression signature and develop a prognostic model as an independent index for overall survival (OS) and disease-free survival (DFS).

## Methods

### Data acquisition

A total of 234 autophagy-related genes were obtained from The Human Autophagy Database (HADb, http://www.autophagy.lu/index.html). RNA-seq data for prostate cancer patients were downloaded from the TCGA data portal (https://tcga-data.nci.nih.gov/tcga/), which contains 485 prostate cancer and 51 adjacent non-tumor tissues. We searched the cBio Cancer Genomics Portal (http://cbioportal.org) to identify the clinical data, including OS and DFS.

### Differentially expressed ARGs and enrichment analysis

Data analysis of differential expression of ARGs between PCa and their non-tumor counterparts was performed using package limma in R, with thresholds of |log_2_ fold change (FC)|> 2 and adjusted P-value < 0.05. Then, we performed gene ontology (GO) enrichment analyses to find the major biological attributes of differential expression ARGs. The visual GO enrichment maps of annotation analysis results were performed by R with the “ggplot2” and “GOplot” packages.

### Construction of prognostic signature based on ARGs

Univariate Cox and multivariate Cox regression analyses were performed to find out the OS-related and DFS-related ARGs in PCa. Then, the OS-related and DFS-related prediction formulas were applied to build prognostic models using package “glmnet” based on the multivariate Cox regression. The survival analysis was assessed by Kaplan–Meier (K–M) methods to compare the high-risk and low-risk groups according to predictive signatures. Finally, the predictive value of prognostic prediction models was evaluated by areas under the curve (AUC) of the receiver-operator characteristic (ROC) curve using package “survivalROC” in R.

### Statistical analysis

All of the statistical tests were done with R 3.3.1 (https://www.r-project.org/) and GraphPad Prism 7 (San Diego, CA, USA). All analyses performed were two-sided, and statistical significance was defined as a P-values < 0.05.

## Results

### Differentially expressed ARGs between prostate cancer and adjacent non-tumor tissues

**Fig. 1:**
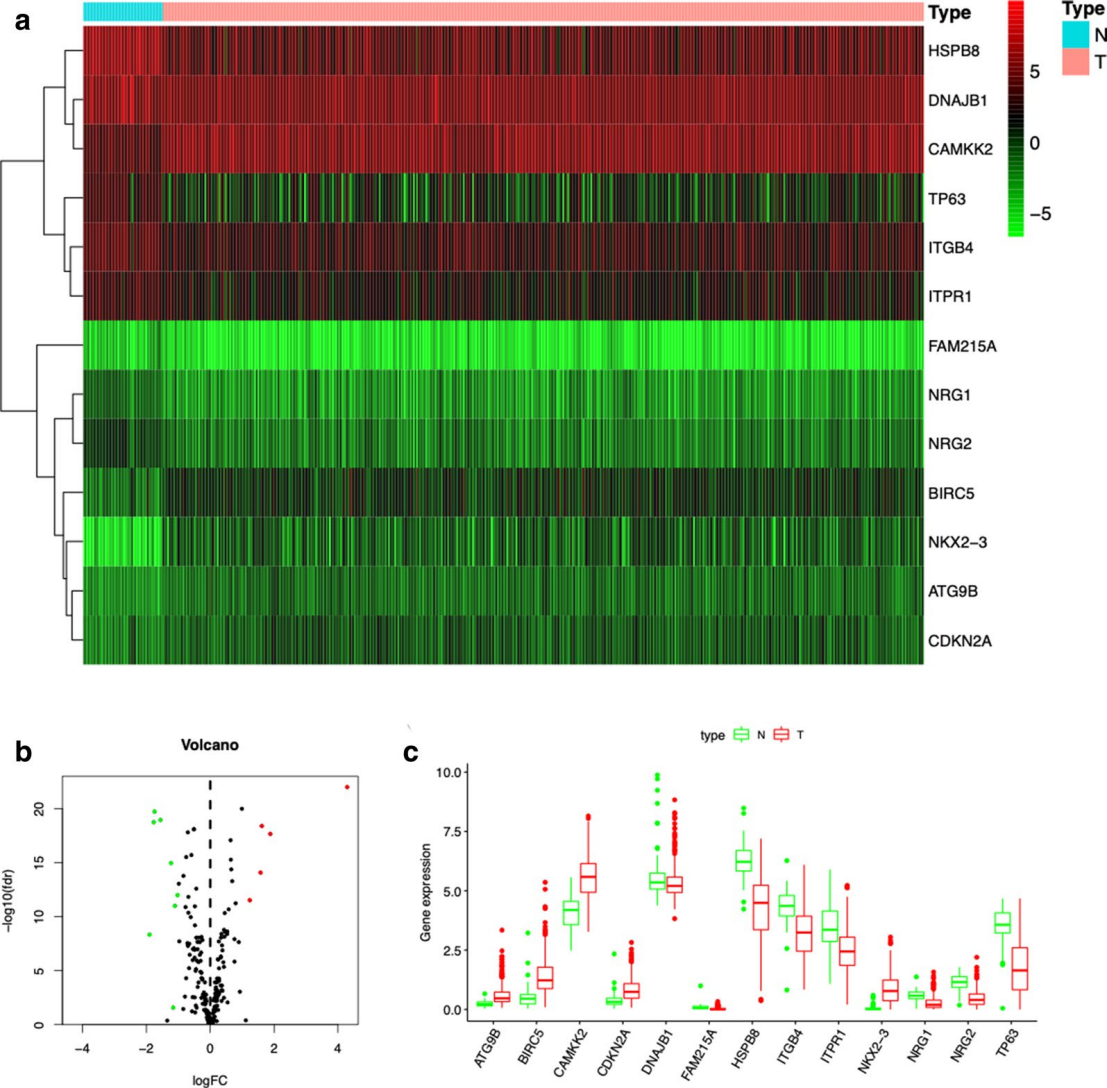
Differentially expressed ARGs between prostate cancer and normal prostate tissues. **a** Heatmap of differentially expressed ARGs. **b** The volcano plot for the 234 ARGs from the TCGA data portal. Red indicates high expression, and green indicates low expression. Black shows that those genes showed no difference between prostate cancer and normal prostate tissues. **c** The expression patterns of 13 differentially expressed ARGs in prostate cancer and paired non-tumor samples. Red and green indicate tumor tissues and normal tissues, respectively

A total of 485 primary PCa patients with RNA-seq data and clinical follow-up information were involved in the present study. Among 234 autophagy-related genes, there were 13 differentially expressed ARGs, including 5 up-regulated (ATG9B, BIRC5, CAMKK2, CDKN2A, and NKX2-3) and 8 down-regulated ARGs (DNAJB1, FAM215A, HSPB8, ITGB4, ITPR1, NRG1, NRG2, and TP63), with thresholds of |log_2_ fold change (FC)| > 2 (Fig. 1a). Then, the volcano plot and box plot were visualized to show the expression pattern of the differentially expressed ARGs between PCa and non-tumor tissues (Fig. 1b, c).

### GO enrichment analysis of differentially expressed autophagy-related genes

**Fig. 2:**
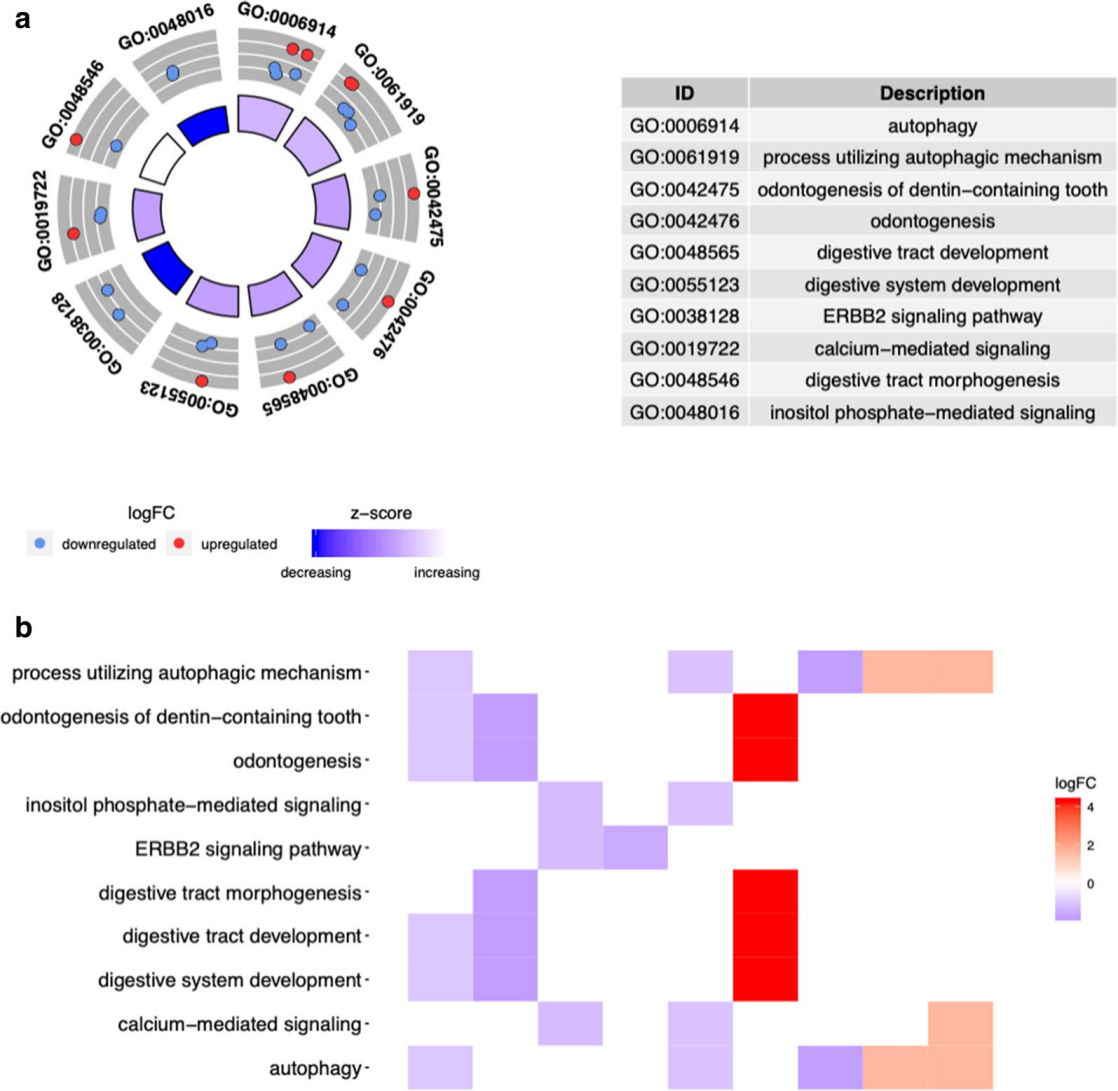
GO enrichment analysis of differentially expressed autophagy-related genes. **a** The outer circle shows a scatter plot for each term of the logFC of the differentially expressed ARGs. **b** Heatmap of the relationship between ARGs and GO enrichment. The color of each block depends on the logFC values

GO enrichment analysis was performed according to the differentially expressed ARGs. According to the results of DAVID, we found that the top enriched GO terms for biological processes were autophagy, process utilizing autophagic mechanism, and odontogenesis of dentin-containing tooth. The heatmap of the relationship between ARGs and GO enrichment analysis was also displayed (Fig. 2a, b).

### Identification of prognosis-related ARGs and construction of prognosis prediction model

A total of 14 ARGs were significantly associated with OS in the univariate Cox regression analysis. Furthermore, in the multivariate Cox regression analysis, five genes including FAM215A, FDD, MYC, RHEB, and ATG16L1 were identified to construct the OS prediction model. OS-related prediction model = (17.20896* expression value of FAM215A) + (4.319028* expression value of FADD) + (0.674838* expression value of MYC) + (1.869633* expression value of RHEB) + (2.071004* expression value of ATG16L1).

**Fig. 3:**
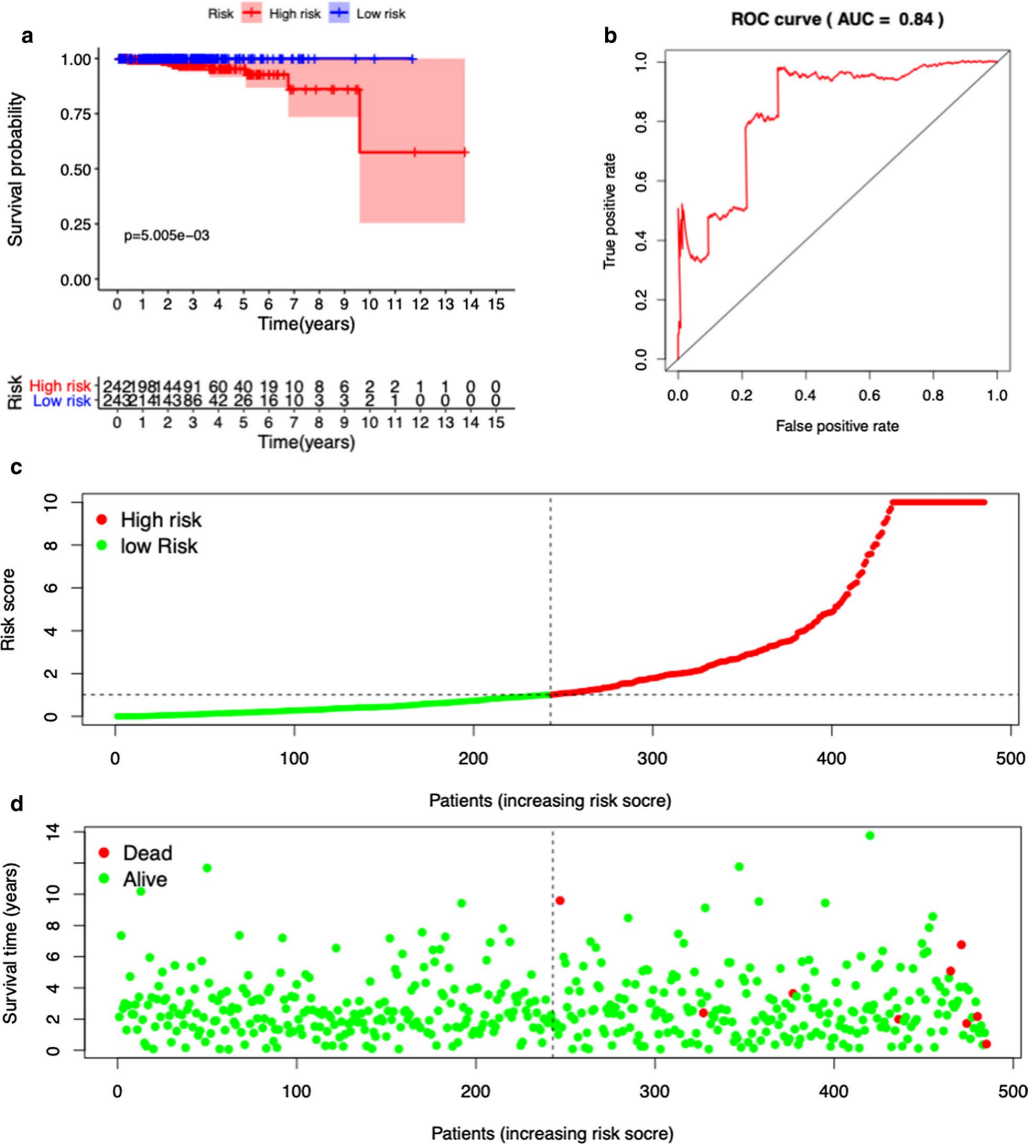
OS-related prognostic model of prostate cancer patients. **a** Kaplan–Meier plot represents that patients in the high-risk group had significantly shorter overall survival than those in the low-risk group. **b** ROC curve of OS-related prognostic model. **c** The prognostic model distribution of prostate patients. **d** The overall survival of patients in the TCGA dataset

We divided the 485 prostate cancer cases into high- and low-risk groups according to the median values of the OS-related prediction model. Kaplan–Meier survival curves showed that low-risk group had a lower mortality rate than high-risk group (HR = 6.391, 95% CI = 1.581–25.840, P < 0.001) (Fig. 3a). The ROC curves of OS-related predictive signatures were demonstrated in Fig. 3b, with AUC of 0.84. Figure 3c, d showed the OS-related prediction model distribution of patients in the TCGA dataset.

**Fig. 4:**
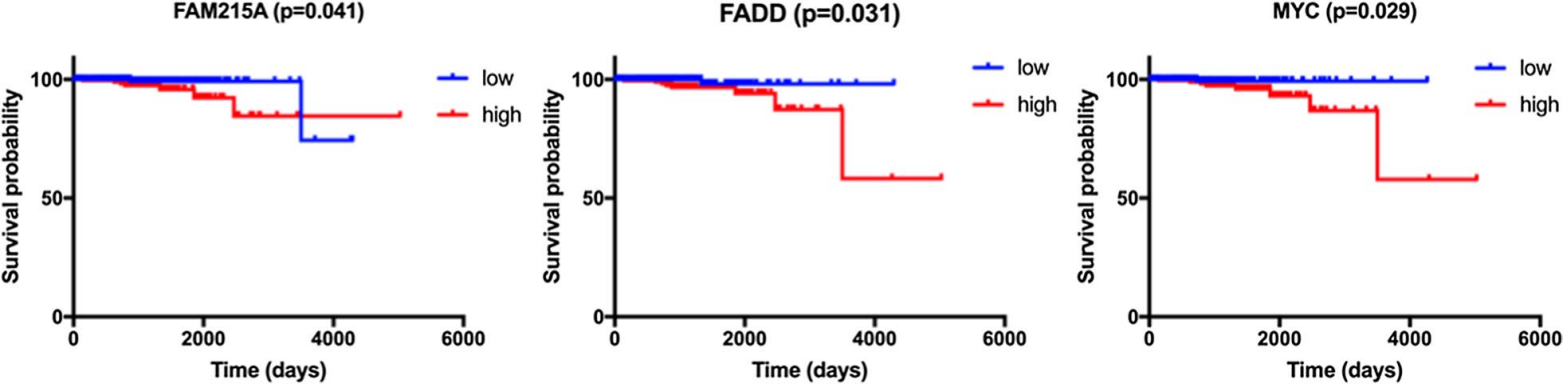
The correlation between ARGs included in OS-related prognostic signature and prostate cancer patients’ survival

According to the median value of the five genes, the high expression level of FAM215A (HR = 4.347, 95% CI = 1.175–16.290, P = 0.041), FADD (HR = 7.009, 95% CI = 1.892–25.960, P = 0.031), and MYC (HR = 7.153, 95% CI = 1.932–26.470, P = 0.029) were significantly associated with worse OS in Kaplan–Meier curves (Fig. 4). However, this association did not hold true of gene ATG16L1(HR = 2.426, 95% CI = 0.653–9.017, P = 0.194) and RHEB (HR = 1.236, 95% CI = 0.335–4.566, P = 0.744) in Kaplan–Meier curves (Additional file 1: Fig. S1).

**Fig. 5:**
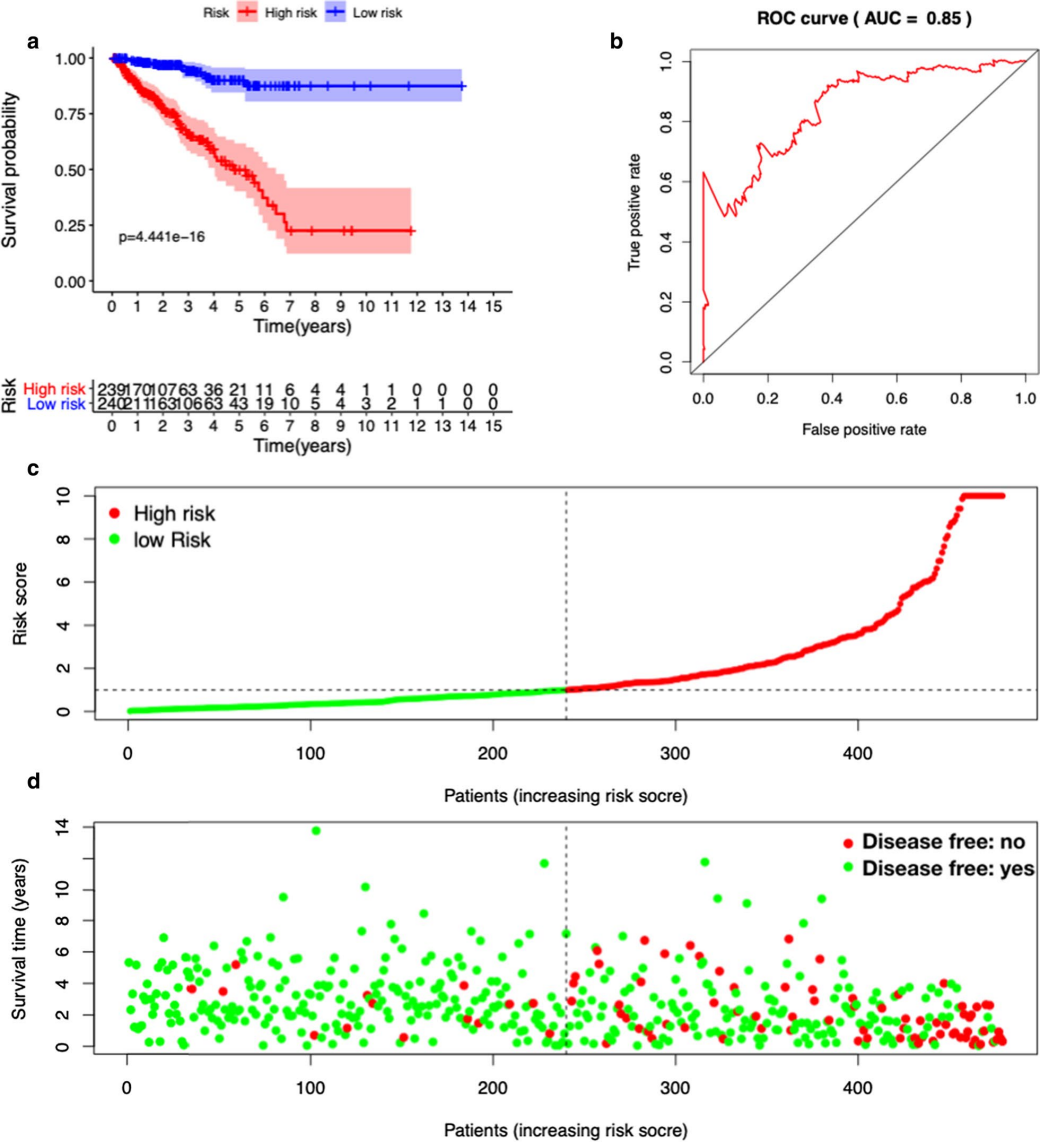
DFS-related prognostic model of prostate cancer patients. **a** Kaplan–Meier plot represents that patients in the high-risk group had significantly shorter overall survival than those in the low-risk group. **b** ROC curve of DFS-related prognostic model. **c** The prognostic model distribution of prostate patients. **d** The disease-free survival of patients in the TCGA dataset

Among 234 autophagy-related genes, a total of 53 ARGs were significantly associated with DFS in the univariate Cox regression analysis. In the multivariate Cox regression analysis, a total of 22 genes were significantly associated with DFS in PCa (Fig. 5a). DFS-related prediction model = (0.97225* expression value of ULK2) + (− 1.74297* expression value of NLRC4) + (− 1.11799* expression value of MAPK1) + (− 1.12182* expression value of ATG4D) + (− 0.73348* expression value of MAPK3) + (1.40252* expression value of ATG2A) + (− 0.49364* expression value of ATG9B) + (− 1.09886* expression value of FOXO1) + (− 0.68955* expression value of PTEN) + (1.80095* expression value of HDAC6) + (− 0.99993* expression value of PRKN) + (0.35846* expression value of HSPB8) + (− 0.51552* expression value of P4HB) + (1.56551* expression value of MAP2K7) + (− 0.96348* expression value of MTOR) + (1.65516* expression value of RHEB) + (0.73934* expression value of TSC1) + (0.27799* expression value of BIRC5) + (1.43484* expression value of RGS19) + (− 0.63037* expression value of RAB24) + (− 0.28580* expression value of PTK6) + (− 1.05312* expression value of NRG2).

We divided the PCa cases into high- and low-risk groups according to the median values of the DFS-related prediction model. Kaplan–Meier survival curves showed that high-risk group had a lower disease-free rate than low-risk group (HR = 7.407, 95% CI = 4.850–11.320, P < 0.001). The ROC curves of OS-related predictive signatures were demonstrated in Fig. 5b, with AUC of 0.85. Figure 5c, d showed the DFS-related prediction model distribution of patients in the TCGA dataset.

**Fig. 6:**
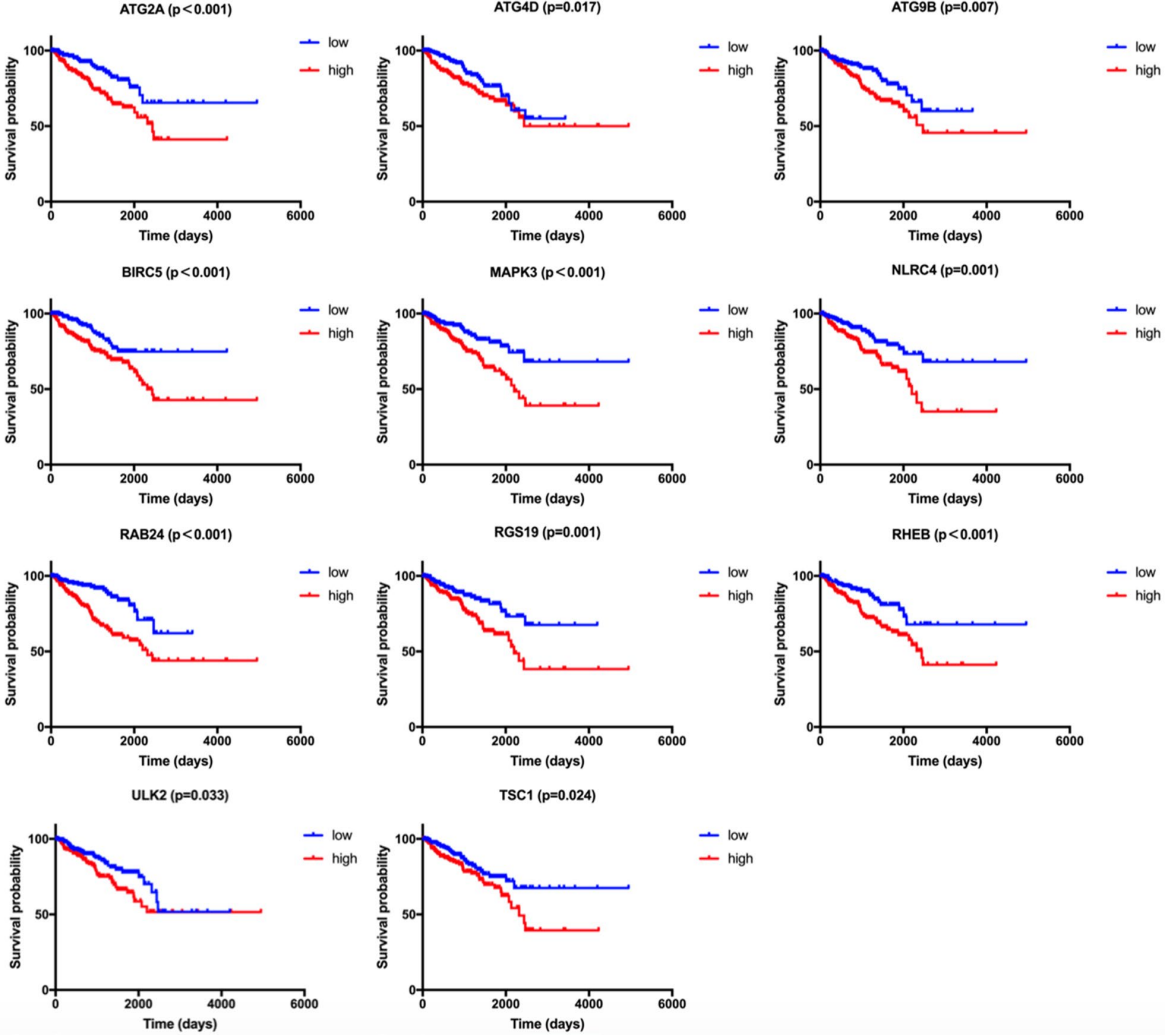
The correlation between ARGs included in DFS-related prognostic signature and prostate cancer patients’ disease-free survival

**Fig. 7:**
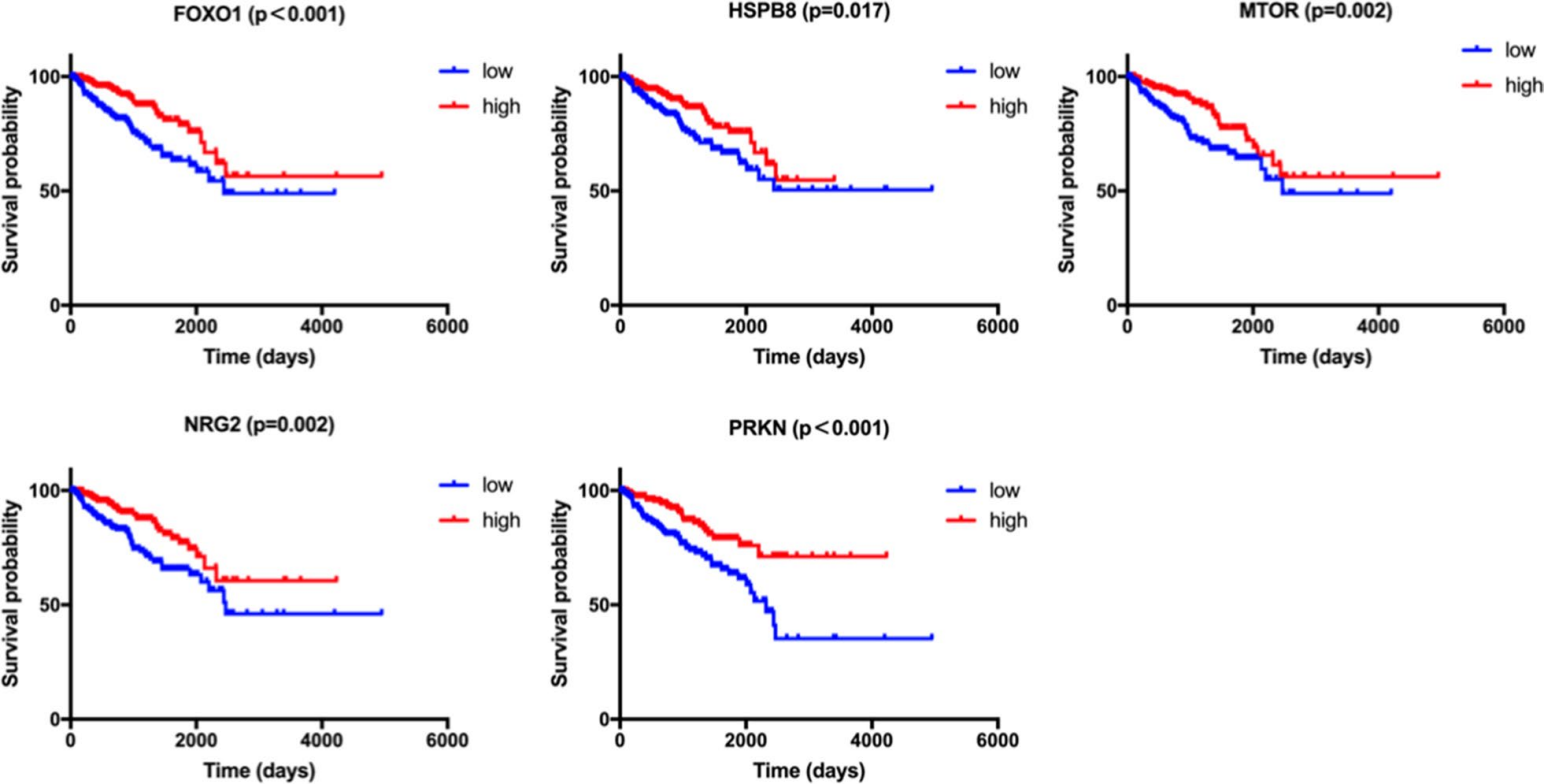
The correlation between ARGs included in DFS-related prognostic signature and prostate cancer patients’ disease-free survival

Among the 22 genes in DFS-related prediction model, high expression of ATG2A (HR = 2.266, 95% CI = 1.492–3.442, P < 0.001), ATG4D (HR = 1.665, 95% CI = 1.096–2.530, P = 0.017), ATG9B (HR = 1.803, 95% CI = 1.187–2.738, P = 0.007), BIRC5 (HR = 2.013, 95% CI = 1.384–3.195, P < 0.001), MAPK3 (HR = 2.148, 95% CI = 1.414–3.263, P < 0.001), NLRC4 (HR = 2.053, 95% CI = 1.352–3.119, P = 0.001), RAB24 (HR = 2.811, 95% CI = 1.851–4.270, P < 0.001), RGS19 (HR = 2.019, 95% CI = 1.329–3.068, P = 0.001), RHEB (HR = 2.137, 95% CI = 1.407–3.245, P < 0.001), ULK2 (HR = 1.579, 95% CI = 1.039–2.399, P = 0.033), and TSC1 (HR = 1.622, 95% CI = 1.067–2.464, P = 0.024) genes were associated with worse prognosis in PCa in Kaplan–Meier curves according to the median values of gene expression (Fig. 6). In addition, high expression of FOXO1 (HR = 2.087, 95% CI = 1.373–3.172, P < 0.001), HSPB8 (HR = 1.673, 95% CI = 1.101–2.541, P = 0.017), MTOR (HR = 1.897, 95% CI = 1.247–2.885, P = 0.002), NRG2 (HR = 1.944, 95% CI = 1.280–2.955, P = 0.002) and PRKN (HR = 2.308, 95% CI = 1.518–3.508, P < 0.001) genes were associated with better prognosis in Kaplan–Meier curves according to the median values of gene expression (Fig. 7). No differences were found between the expression level of HDAC6 (HR = 1.392, 95% CI = 0.913–2.123, P = 0.116), MAP2K7 (HR = 1.379, 95% CI = 0.908–2.094, P = 0.133), MAPK1 (HR = 1.426, 95% CI = 0.939–2.167, P = 0.095), P4HB (HR = 1.501, 95% CI = 0.988–2.280, P = 0.058), PTK6 (HR = 1.338, 95% CI = 0.881–2.032, P = 0.174), and PTEN (HR = 1.324, 95% CI = 0.872–2.010, P = 0.191) and disease-free survival (Additional file 2: Fig. S2).

### The relationships between clinicopathological parameters and prognosis-related ARGs and prognosis-related prediction model

**Fig. 8:**
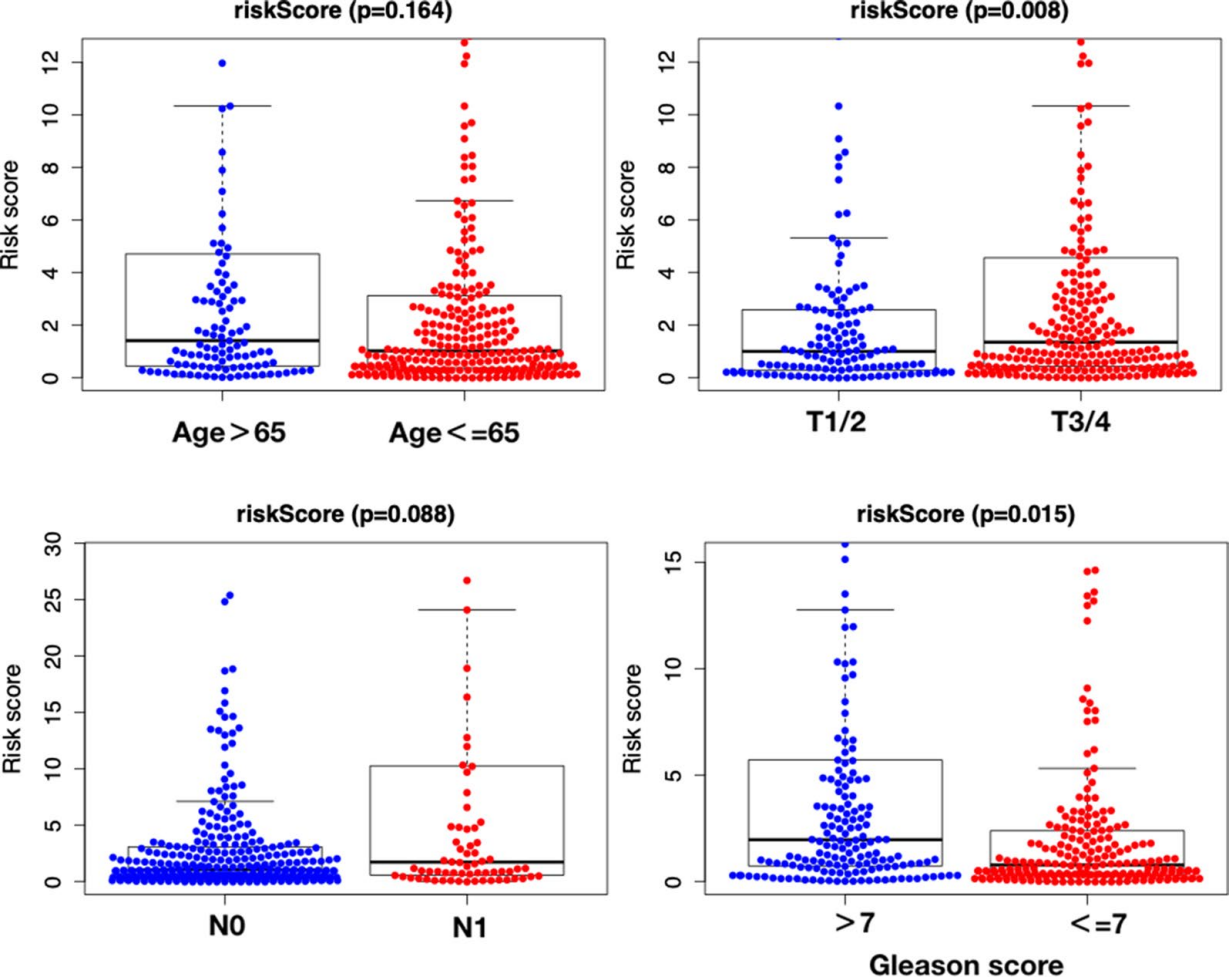
The clinicopathological significance of OS-related prognostic model in prostate cancer

**Fig. 9:**
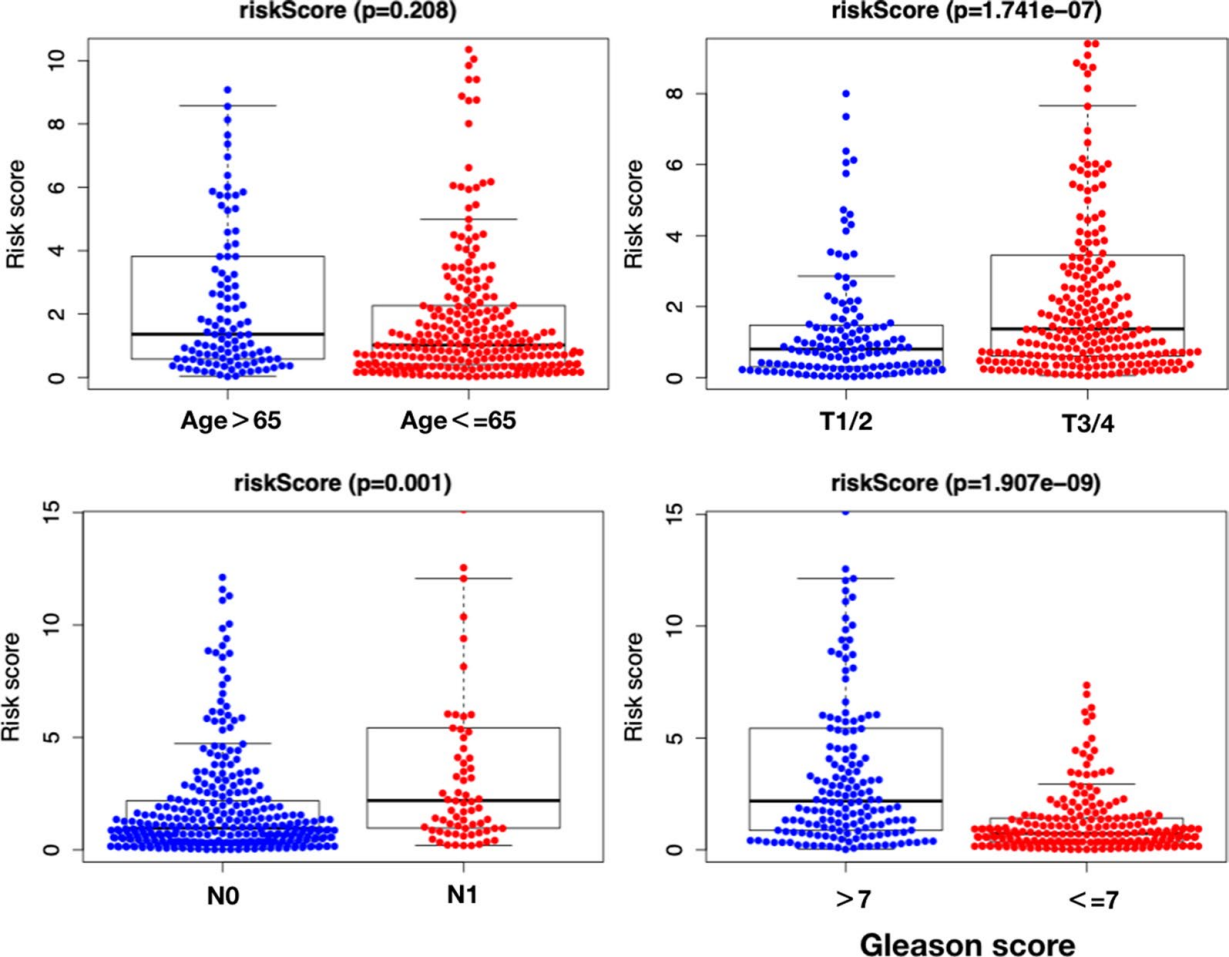
The clinicopathological significance of DFS-related prognostic model in prostate cancer

The OS-related prediction model values were higher in T3-4 than in T1-2 (P = 0.008), and higher in Gleason score > 7 than  ≤ 7 (P = 0.015). No difference of OS-related prediction model values was observed between age > 65 than age ≤ 65 (P = 0.164), or N0 stage and N1 stage (P = 0.088) (Fig. 8). The DFS-related prediction model values were higher in T3-4 than in T1-2 (P < 0.001), higher in N1 than in N0 (P = 0.001), and higher in Gleason score  > 7 than  ≤ 7 (P < 0.001). No difference of DFS-related prediction model values was observed between age > 65 than age ≤ 65 (P = 0.208) (Fig. 9).

Among 485 primary PCa patients in the present study, only two of them had distant metastasis. Therefore, the relationship between the M status and prediction model has not been analyzed.

## Discussion

Much evidence has indicated that autophagy participates in multiple signaling pathways to play a role in the proliferation and invasion of prostate cancer [9, 13]. Additionally, Blessing et al. demonstrated that four core autophagy genes (ATG4B, ATG4D, ULK1, and ULK2) regulate androgen receptor (AR) activity, thereby affecting the biological behavior of prostate cancer [14].

In the present study, we mined the expression profiles of ARGs from the TCGA database and aimed to analyze the association between ARGs and the prognosis of prostate cancer patients. Firstly, we screened 13 differentially expressed ARGs between prostate cancer and non-tumor tissues, that many of them play a role in the biological processes by GO term analysis.

Then, a total of 14 OS-related ARGs were found in the univariate Cox regression analysis. Further multivariate Cox regression analysis was performed to determine five OS-related ARGs (FAM215A, FDD, MYC, RHEB, and ATG16L1) and construct the OS-related prediction model, which could be an independent prognostic indicator for PCa patients.

Handle et al. found that MYC activity is closely related to AR, which regulates the growth of anti-androgen resistant cell lines [15]. Kobayashi et al. demonstrated that RHEB mRNA and protein expression was higher in more aggressive prostate cancer cell lines (PC3 and DU145) compared with the less aggressive LNCaP. Moreover, inhibition of RHEB can lead to the suppressed proliferation of prostate cancer cell lines [16]. Previous research analyzed the relationship between genetic variants of the autophagy pathway and clinical outcomes in 458 prostate cancer patients, which indicated that high expression of ATG16L1 was correlated with lower tumor aggressiveness and favorable prognosis [17]. Fu et al. reported that high expression of FAM215A was associated with low tumor grades, early disease stages, and favorable overall survival in epithelial ovarian cancer [18]. FDD is a component of FMNL3, and high expression of FMNL3 associated with cancer cell migration, invasion, and unfavorable prognosis in tongue squamous cell carcinoma [19]. However, the function of FAM215A and FDD gene has not been reported in prostate cancer, indicating that functional studies on these genes may help us to understand the prognosis-related biological behavior of bladder cancers more accurately.

In the present study, a total of 22 ARGs were significantly associated with DFS of PCa in multivariate Cox regression analysis, including ULK2, NLRC4, MAPK1, ATG4D, MAPK3, ATG2A, ATG9B, FOXO1, PTEN, HDAC6, PRKN, HSPB8, P4HB, MAP2K7, MTOR, RHEB, TSC1, BIRC5, RGS19, RAB24, PTK6, and NRG2. Previous research has shown that ULK2 and ATG4D were hub autophagy genes, which are necessary for maximal androgen-mediated autophagy and cell proliferation and also associated with poor prognosis in PCa [14]. Li et al. demonstrated that MAPK1 plays an important role in regulating cancer cell invasion and metastasis in vitro and in vivo [20]. Forkhead box transcription factor-1 (FOXO1) is a tumor suppressor that is downregulated in human prostate cancer, which acts as a repression target of EZH2 and an essential mediator of EZH2 inhibition-induced cell death [21]. PTEN is one of the most commonly altered tumor suppressor genes in prostate cancer, which negatively regulates the PI3K/AKT/mTOR signaling pathway. PTEN deletion is associated with poorer cancer-specific outcomes, increasing stage, and higher Gleason score [22]. Chuang et al. suggested that HDAC6 has anti-cancer activity in prostate cancer, which participates in regulating the cRaf-PP1-ERK signaling pathway and contributing to M phase cell-cycle transition [23]. Many studies have shown that multiple oncogenes promote PCa cell proliferation, migration, invasion, and inhibiting apoptosis through activating the PI3K-AKT-mTOR signaling pathway [24]. Chen et al. found that the high levels of NPRL2 gene expression in prostate cancer cells promote resistance to EVS (an inhibitor of the mTOR) by enhancing autophagy [25]. In addition, TSC1 was significantly associated with DFS in PCa, which is an essential component of the PI3K/AKT/mTOR signaling pathway [26]. Among the 22 DFS-related ARGs, except for those mentioned above, other ARGs are either poorly investigated or have not been reported, which means our findings suggested further research for them is imperative.

The OS and DFS-related prediction model values were both associated with the T stage and Gleason score in PCa patients, higher in T3/4 than in T1/2, and higher in Gleason score > 7 than ≤ 7. Patients with T3 or T4 stage are also known as locally advanced prostate cancer. Krimphove et al. reported that PCa with T3 or T4 had a worse overall survival [27]. Meanwhile, the Gleason score is the sum of the two most common grade patterns in PCa, which act as the single most potent predictor of PCa outcomes [28]. DFS-related prediction model values were higher in N1 than in N0. N1 is defined as regional lymph node metastasis in AJCC/UICC N category. Jin et al. proposed lymph node ratio (LNR) and log odds of metastatic lymph node (LODDS) staging may be better predictors of overall survival than the AJCC/UICC N category [29]. Accumulating evidence indicates that the characteristics of gene expression are significantly correlated with the patient’s adverse clinical parameters.

## Conclusion

In conclusion, our current study assessed the autophagy-related genes expression profiles based on the TCGA database. It proposed an OS-related and a DFS-related prediction model, which had good efficacy in predicting the OS and DFS of PCa patients. A total of five OS-related ARGs (FAM215A, FDD, MYC, RHEB, and ATG16L1) and twenty-two DFS-related ARGs (ULK2, NLRC4, MAPK1, ATG4D, MAPK3, ATG2A, ATG9B, FOXO1, PTEN, HDAC6, PRKN, HSPB8, P4HB, MAP2K7, MTOR, RHEB, TSC1, BIRC5, RGS19, RAB24, PTK6, and NRG2) were identified. These results showed that the autophagy-related genes signature may act as a promising prognostic molecular biomarker in PCa. Moreover, further research of these hub genes may contribute to molecular targeted therapy of prostate cancer.

## Supplementary information

**Additional file 1: Figure S1.** The correlation between ATG16L1 and RHEB and OS in Kaplan–Meier curves.

**Additional file 2: Figure S2.** The correlation between HDAC6, MAP2K7, MAPK1, P4HB, PTK6, and PTEN and DFS in Kaplan–Meier curves.

## Data Availability

Not applicable.

## Abbreviations

PCa: Prostate cancer
ARGs: Autophagy-related genes
TCGA: The genomic data commons data portal and the cancer genome atlas database
GO: Gene ontology database
ROC curve: Receiver operating characteristic curve
AUC: Area under curve
OS: Overall survival
DFS: Disease-free survival
