# Supplementary figures and images for: Development of an autophagy-related gene expression signature for prognosis prediction in prostate cancer patients
Source: J Transl Med. 2020 Apr 7;18:160. doi: 10.1186/s12967-020-02323-x (PMC7137440; doi:10.1186/s12967-020-02323-x)

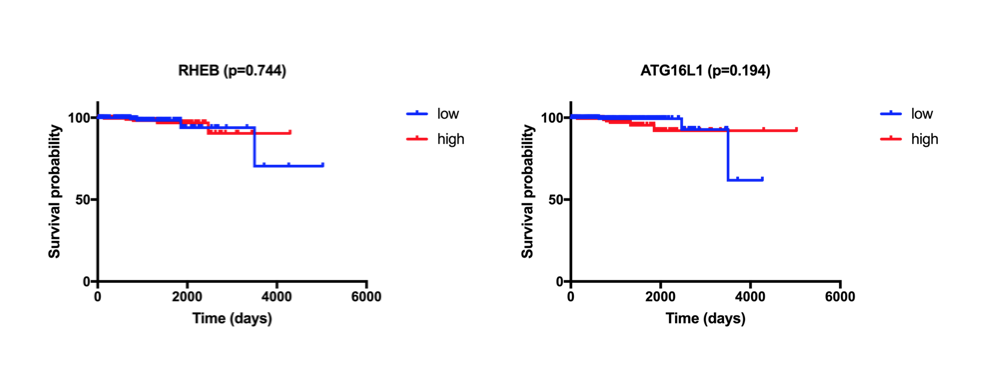

Supplement: Supplementary file 1 — Additional file 1: Figure S1. The correlation between ATG16L1 and RHEB and OS in Kaplan–Meier curves. [file 12967_2020_2323_MOESM1_ESM.tiff]

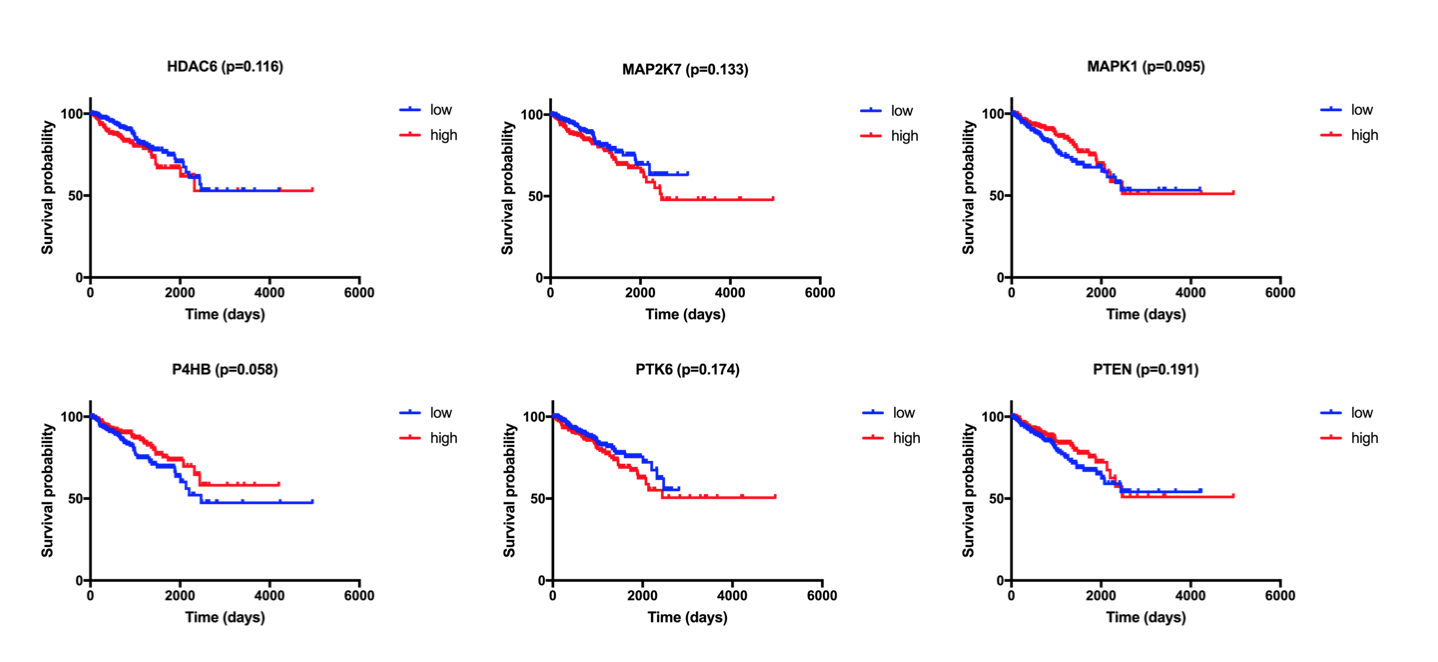

Supplement: Supplementary file 2 — Additional file 2: Figure S2. The correlation between HDAC6, MAP2K7, MAPK1, P4HB, PTK6, and PTEN and DFS in Kaplan–Meier curves. [file 12967_2020_2323_MOESM2_ESM.tiff]
